# Medication profiling in women with type 1 diabetes highlights the importance of adequate, guideline-based treatment in low-risk groups

**DOI:** 10.1038/s41598-023-44695-2

**Published:** 2023-10-19

**Authors:** Raija Lithovius, Stefan Mutter, Erika B. Parente, Ville-Petteri Mäkinen, Erkka Valo, Valma Harjutsalo, Per-Henrik Groop

**Affiliations:** 1 Folkhälsan Institute of Genetics, Folkhälsan Research Center, Helsinki, Finland; 2 Department of Nephrology, University of Helsinki and Helsinki University Hospital, Helsinki, Finland; 3 Research Program for Clinical and Molecular Metabolism, Faculty of Medicine, University of Helsinki, Helsinki, Finland; 4 Systems Epidemiology, Faculty of Medicine, University of Oulu, Oulu, Finland; 5 Research Unit of Population Health, Faculty of Medicine, University of Oulu, Oulu, Finland; 6 Chronic Disease Prevention Unit, National Institute for Health and Welfare, Helsinki, Finland; 7 Department of Diabetes, Central Clinical School, Monash University, Melbourne, VIC Australia; 8 Folkhälsan Institute of Genetics, Folkhälsan Research Center, Biomedicum Helsinki, Helsinki University, Haartmaninkatu 8 [C318b], PO Box 63, 00014 Helsinki, Finland

**Keywords:** Endocrinology, Nephrology

## Abstract

Effective treatment may prevent kidney complications, but women might be underprescribed. Novel, data-driven insights into prescriptions and their relationship with kidney health in women with type 1 diabetes may help to optimize treatment. We identified six medication profiles in 1164 women from the FinnDiane Study with normal albumin excretion rate based on clusters of their baseline prescription data using a self-organizing map. Future rapid kidney function decline was defined as an annual estimated glomerular filtration rate (eGFR) loss > 3 ml/min/1.73 m^2^ after baseline. Two profiles were associated with future decline: Profile *ARB* with the highest proportion of angiotensin receptor blockers (odds ratio [OR] 2.75, *P* = 0.02) and highly medicated women in profile *HighMed* (OR 2.55, *P* = 0.03). Compared with profile *LowMed* (low purchases of all)*,* profile *HighMed* had worse clinical characteristics, whereas in profile *ARB* only systolic blood pressure was elevated. Importantly, the younger women in profile *ARB* with fewer kidney protective treatments developed a rapid decline despite otherwise similar baseline characteristics to profile *ACE & Lipids* (the highest proportions of ACE inhibitors and lipid-modifying agents) without a future rapid decline. In conclusion, medication profiles identified different future eGFR trajectories in women with type 1 diabetes revealing potential treatment gaps for younger women.

## Introduction

Type 1 diabetes is a common chronic disease, which usually manifests early in life, and therefore, the affected individuals are at high lifetime risk to develop diabetic complications^1^. One-third of these individuals develop diabetic kidney disease (DKD), which is the leading cause of kidney failure in developed countries, requiring dialysis and kidney transplantation^2, 3^. Kidney function, assessed by the estimated glomerular filtration rate (eGFR), declines progressively along with ageing^4^. Although diabetes, high blood pressure (BP) and dyslipidemia speed up the loss of kidney function, the overall reduction of the eGFR varies between individuals^5^. The concept of a rapid decline in the eGFR, defined as an annual eGFR loss > 3 ml/min/1.73 m^2^^6^, has been proposed as an early phenotype of kidney disease, which is associated with a high risk of cardiovascular disease (CVD)^7, 8^.

Although pharmacological interventions play a fundamental role in preventing and treating diabetic co-morbidities, kidney function may decline rapidly in some individuals despite kidney-protective therapies^9^. Moreover, some individuals who might benefit from pharmacological therapies may not receive the optimal treatment at the right time due to misclassification of their risk of kidney disease^9, 10^. One example is the sex disparity in the cardio- and kidney-protective treatment in individuals with diabetes^11–13^. Women’s risk-reducing measures are often underestimated, and usually women report less frequent use of protective medications than men^12^. Of note, the cardio-protective effect of the female sex is lost in the presence of type 1 diabetes in contrast to people without diabetes^14^, and the CVD risk is even 20-fold higher in women with diabetes compared to control subjects^15^. Therefore, a major challenge is the early identification and timely initiated organ-protective treatment of high-risk individuals^16^, which consequently may help to prevent or slow down a rapid decline of kidney function, and possibly improve kidney and CVD outcomes^17^.

Considering that there is a lack of relevant information regarding medication prescriptions and their relationship with kidney outcomes in women with type 1 diabetes, in this study, we created medication profiles for women with type 1 diabetes and estimated, whether any of these profiles were associated with a future rapid decline of eGFR. Our data-driven method^18^ allows combining the prescription information of a large number of pharmacological subgroups, that cover the entire range of pharmacological treatments for each woman in the study. Therefore, our results present important, data-driven insights into the complex diversity of concurrent medication use by investigating how characteristic profiles of medication use associate with kidney outcomes. This may further help to optimize prescriptions for women with type 1 diabetes before any decline of kidney function.

## Methods

### The FinnDiane study cohort

The current study is part of the ongoing, prospective, nationwide, multicenter Finnish Diabetic Nephropathy (FinnDiane) study, designed to identify mechanisms and risk factors of diabetic complications in individuals with type 1 diabetes, with an emphasis on DKD. A more detailed description of the study protocol has been reported previous^19, 20^. In brief, the study was launched 1997 and currently includes about 5400 adults with type 1 diabetes who were recruited from more than 90 hospitals and health care centers throughout Finland (see Supplementary Table S1). In addition, a prospective phase was launched in 2004, covering about 1600 follow-up visits (arranged every fifth year). All adult individuals with type 1 diabetes were recruited regardless of the duration of diabetes or presence of complications, and therefore had an equal probability to participate. Both baseline and prospective visits were carried out according to the same protocol. Type 1 diabetes was defined by diabetes onset below 40 years of age and initiation of insulin treatment within one year. As part of the FinnDiane protocol, details of the clinical characteristics of the individuals were obtained from medical records by the attending physician using a standardized questionnaire. Each participant also completed a detailed questionnaire on lifestyle, smoking habits and family history. In addition, data were collected from the medical records and various national registers, such as the Care Register for Health Care, the Causes of Death Register and the Drug Prescription Register. Moreover, early morning blood samples were collected for analysis of HbA1c, lipids and creatinine and urine samples for the measurement of albumin excretion rate (AER) amongst other urinary markers. Normal urinary AER was defined by an AER < 30 mg/24 h or < 20 μg/min or an albumin-to-creatinine ratio < 3.5 mg/mmol in two out of three consecutive measurements^21^.The baseline data for the individuals included in this study were collected between 1995 and 2013. Pregnancy-related visits (ICD-10 codes O00-O99) were obtained from the Care Register for Health Care and were recorded one-year before and after the baseline. Treatment targets of BP, triglycerides and LDL-cholesterol were based on the American Diabetes Association diabetes guidelines^22^. The study protocol was approved by the Ethics Committee of the Helsinki and Uusimaa Hospital District, and the study was carried out in accordance with the Declaration of Helsinki. Written informed consent was obtained from each participant.

### eGFR slopes

During a median follow-up of 11.3 (IQR 7.5–13.6) years, the eGFR slopes were calculated by fitting individual linear regression lines of consecutive eGFR values reported during the follow-up separately for each woman. We collected eGFR values from baseline, prospective study visits and medical records. The Chronic Kidney Disease Epidemiology Collaboration (CKD-EPI) equation was used to calculate eGFR^23^. Women with fewer than three eGFR values and less than two years of follow-up to calculate slopes were excluded from the study. With this setup, the impact of acute falls in eGFR caused by a potential initiation of renin–angiotensin–aldosterone system (RAAS) inhibition were minimised. The women were followed-up from the baseline until the last eGFR value, initiation of dialysis or kidney transplantation or until the end of 2015. The outcome measure of the study was a rapid decline of kidney function defined as an eGFR slope steeper than − 3 ml/min/1.73 m^2^ per year^6^. Upon visual inspection of all slopes, we did not find any impact of RAAS initiation on our outcome measure.

### Baseline medication profiles

By linkage to the Finnish Drug Prescription Register (DPR), all purchases of outpatient prescription drugs were obtained until the end of 2015. Medications were recorded using the ATC classification system^24^, which consists of 5 levels starting from 14 anatomical main groups on the 1st level down to the individual chemical substance on the 5th level. To ensure the generalization ability of our models, we used the 3rd level indicating pharmacological subgroups. Insulins and analogues (ATC code A10A) and any pharmacological subgroups found in less than 2% of prescriptions at baseline were excluded from the study leaving us with 37 pharmacological subgroups (Supplementary Table S2). For each woman and each pharmacological subgroup, a dichotomous indicator for at least one purchase event (i.e., each time a person purchases prescribed medication from a pharmacy) within the baseline window (180 days before or after the baseline visit) was used for the construction of medication profiles. A baseline medication profile was defined as a cluster of women with similar purchase patterns and was derived in a two-step approach.

**Figure 1:**
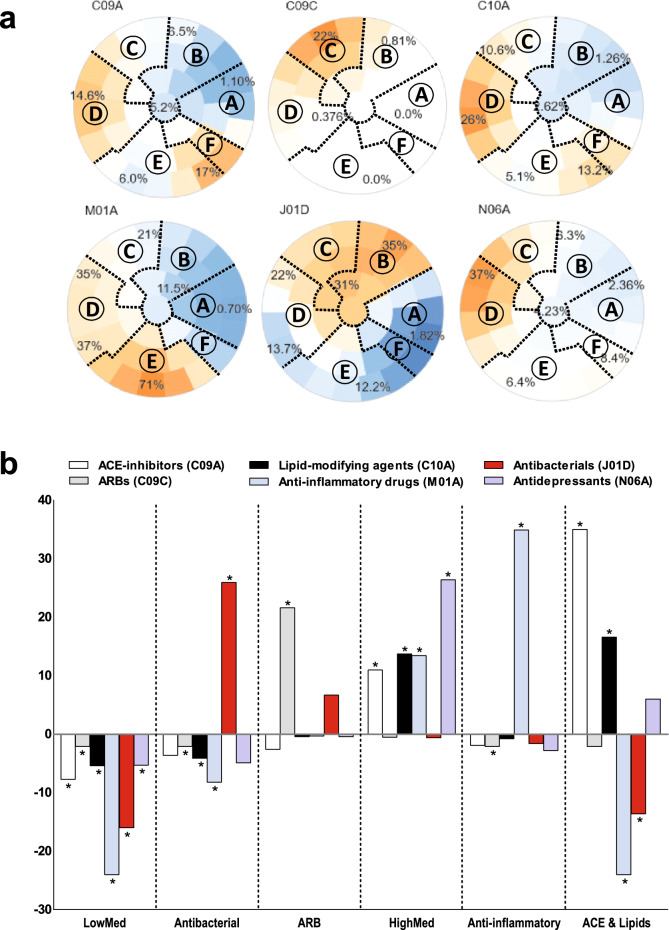
(**A**) Self-organizing maps (SOM) and six selected pharmacological subgroups (based on the 3rd level ATC classification), either known to be protective or harmful for the kidneys and which showed a clear patterns in deviation from the study average in purchases of prescription medications at each selected pharmacological subgroups. These selected subgroups were ACE inhibitors [C09A], angiotensin II receptor blockers, ARBs [C09C], lipid-modifying agents [C10A], anti-inflammatory drugs [M01A], antibacterials [J01D], and antidepressants [N06A]. In the coloring scale red refers higher and blue lower deviation from the whole cohort’s average. The intensity of the color shows how much regional variation deviates from the null model. If the observed pattern is stronger than could be expected by change, colors are bright indicating strong impact, while light colors indicates that there is no clear pattern. Moreover, the numerical values on the six map colorings indicate the local mean value of purchasers for that particular subarea. Based on the map colorings, experts’ knowledge was used in setting and deciding all subgroup boundaries (dashed lines). Six baseline medication profiles were identified: *LowMed* (region A), *Antibacterial* (region B), *ARB* (region C), *HighMed* (region D), *Anti-inflammatory* (region E) *and ACE & Lipids* (region F). Each profile represents specific subsets of women that share the same medication pattern at baseline. (**B**) Bars show how proportions of the six medication subgroups differed from the study average at each medication profile. The astrisks (*) denotes the statistical significance of *P* < 0.05, compared with the the study average.

First, in a data-driven step, we created statistically validated, two-dimensional, so-called map colorings using a self-organizing map (SOM)^18^. This SOM-based approach has previously been successfully used in our cohort and others^25–28^. SOM-based clustering fits our analysis particularly well as the inherent number of different medication profiles is unknown, and there are many pharmacological subgroups^18, 29^. A more detailed introduction to the SOM-based framework is available elsewhere^18^. Briefly, the SOM framework creates a two-dimensional representation, that is, a “map” of the women in our study. On the map, women who are far apart are different with respect to prescribed medications, whereas women in the same map neighborhood share a similar overall medication profile. On top of this map, the software estimates statistics according to how many prescriptions containing a certain pharmacological subgroup were observed in a map neighborhood. In the second step, based on selected map colorings representing clinically relevant pharmacological subgroups that showed a clear deviation from the whole cohort's averages, and that are either known to be protective or harmful for the kidneys (Fig. 1), domain experts (RL, SM and EBP) defined boundaries on the map and chose six characteristic so-called medication profiles. These medication profiles represent specific subsets of women, that share the same medication pattern at baseline. Supplementary Fig. 1 shows a schematic illustration of the procedure to create medication profiles.

### Prescription follow-up

In further analysis, for the first five years of follow-up, we recorded from the DPR for each woman whether she had a prescription for a RAAS inhibitor or lipid-lowering medication during each follow-up year.

### Statistical methods

Continuous data were described with mean ± s.d. for normally distributed variables, and as median with interquartile range (IQR) for non-normally distributed data. Differences between the profiles were tested with t-test or Wilcoxon signed rank test, for normally and non-normally distributed variables, respectively. Binary variables were expressed as frequency and differences in distributions were tested with Pearson *χ*^2^ test or two-tailed Fisher exact test, as appropriate. In addition, multivariable logistic regression analyses, adjusted for covariates with clinical relevance (i.e., baseline age, systolic BP, waist-to-height ratio, triglycerides, LDL-cholesterol, glycated hemoglobin [HbA_1c_], smoking, and eGFR), were performed to estimate associations between the medication profiles and rapid decline of kidney function. A p-value of < 0.05 was considered statistically significant. All analyses were performed in R statistical software version 4.0.3^30^.

## Results

### Characteristics of six medication profiles

In the final study cohort, we included 1164 women who had normal AER at the FinnDiane baseline visit and who fulfilled criteria for calculating eGFR slopes. The mean age of the women was 36.4 ± 12.2 years and the mean duration of diabetes 19.5 ± 12.0 years. Using a data-driven framework, we detected six distinct baseline medication profiles (Fig. 1A,B, Supplementary Table S3). The medication profile *LowMed* was characterized by a lower proportion of purchases in all six pharmacological subgroups at baseline compared with the study average. In the profile *Antibacterial*, the percentage of antibacterial medication purchases was the highest. In the profile *ARB,* the proportion of ARB purchases was the highest. In the profile *HighMed*, purchases in all six pharmacological subgroups were higher or did not differ from the study average. The profile *Anti-inflammatory* had the highest proportion of anti-inflammatory and antirheumatic medication purchases. Finally, in the profile *ACE & Lipids*, the proportions of ACE inhibitors and lipid-modifying drug purchases were the highest.

Table 1 shows the baseline clinical characteristics of the women with respect to these six medication profiles. Using the profile *LowMed* as the reference group*,* women with the profile *Antibacterial* had a longer duration of diabetes and lower HDL cholesterol, but no difference in age. In the profile *ARB*, there were no differences in age or duration of diabetes, but the systolic BP was higher. Moreover, in the profile *HighMed*, these women were older, had a longer duration of diabetes, higher systolic BP, lower eGFR, and there was a higher percentage of individuals with a history of CVD. In the profile *Anti-inflammatory*, these women were older, had a longer duration of diabetes, higher systolic BP and triglycerides, as well as a marginally lower eGFR. Finally, in the profile *ACE & Lipids*, these women were older, with a longer duration of diabetes, higher total, and LDL cholesterol, and both higher systolic and diastolic BP. All, except the profile *ARB,* had a higher waist-to-height ratio, compared with the reference group. No differences in glycemic control or history of smoking were observed between the groups in comparison with profile *LowMed*.

**Table 1:** Baseline characteristics of women with type 1 diabetes according to the six medication profiles.

Profile	*LowMed*	*Antibacterial*	*p* value	ARB	*p* value	*HighMed*	*p* value	*Anti-inflammatory*	*p* value	*ACE & Lipids*	*p* value
N (%)	329 (28.3)	241 (20.7)		97 (8.3)		123 (10.6)		292 (25.1)		82 (7.0)	
Age (years)	33.4 ± 11.5	35.0 ± 12.0	0.1	35.4 ± 13.4	0.2	44.3 ± 12.2	< 0.0001	36.5 ± 11.2	0.001	40.9 ± 10.9	< 0.0001
Duration of diabetes (years)	17.1 ± 10.9	19.3 ± 11.1	0.02	18.4 ± 13.3	0.4	26.4 ± 13.9	< 0.0001	19.3 ± 11.4	0.01	21.7 ± 11.7	0.002
Age at onset of diabetes (years), median (IQR)	14.9 (9.9–22.2)	13.9 (9.5–22.1)	0.5	14.2 (10.0–23.7)	0.8	14.9 (9.3–26.4)	0.3	15.0 (10.9–24.2)	0.2	17.9 (10.9–25.7)	0.02
HbA1c (%)	8.3 ± 1.5	8.4 ± 1.5	0.5	8.4 ± 1.3	0.5	8.4 ± 1.2	0.3	8.4 ± 1.4	0.4	8.1 ± 1.4	0.4
HbA1c (mmol/mol)	(67 ± 17)	(68 ± 16)	0.5	(68 ± 14)	0.5	(68 ± 13)	0.3	(68 ± 16)	0.4	(65 ± 15)	0.4
Systolic BP (mmHg)	124 ± 15	126 ± 15	0.1	128 ± 17	0.02	132 ± 16	< 0.0001	127 ± 16	0.002	133 ± 18	< 0.0001
Diastolic BP (mmHg)	76 ± 9	77 ± 8	0.1	77 ± 9	0.2	76 ± 8	0.5	78 ± 9	0.01	80 ± 10	0.001
Waist-to-height ratio	0.47 ± 0.05	0.49 ± 0.07	0.002	0.47 ± 0.05	0.1	0.50 ± 0.07	< 0.0001	0.49 ± 0.06	0.002	0.49 ± 0.07	0.02
Total cholesterol (mmol/l)	4.79 ± 0.76	4.80 ± 0.90	1.0	4.97 ± 0.91	0.08	4.92 ± 0.80	0.1	4.85 ± 0.80	0.3	5.08 ± 0.95	0.01
LDL cholesterol (mmol/l)	2.89 ± 0.73	2.94 ± 0.84	0.4	2.96 ± 0.84	0.5	2.99 ± 0.78	0.2	2.94 ± 0.78	0.4	3.11 ± 0.94	0.05
HDL cholesterol (mmol/l)	1.50 ± 0.39	1.41 ± 0.37	0.005	1.54 ± 0.39	0.4	1.48 ± 0.36	0.5	1.48 ± 0.40	0.5	1.54 ± 0.47	0.5
Triglycerides (mmol/l), median (IQR)	0.83 (0.65–1.11)	0.88 (0.69–1.18)	0.07	0.88 (0.68–1.26)	0.1	0.87 (0.69–1.17)	0.1	0.92 (0.70–1.28)	0.009	0.88 (0.65–1.15)	0.5
History of smoking, %	35.6	34.6	0.9	33.7	0.8	41.2	0.3	41.8	0.1	33.3	0.8
Baseline FinnDiane visit (years), median (IQR)	2000 (1999–2002)	2000 (1998–2002)	0.008	2001 (2000 –2002)	0.2	2001 (1999 –2003)	0.3	2000 (1999–2002)	1.0	2000 (1999–2002)	0.7
eGFR (ml/min/1.73 m^2^), median (IQR)	104 (90–117)	103 (90–116)	0.6	103 (87–116)	0.4	96 (85–108)	0.0003	101 (86–112)	0.02	102 (84–111)	0.1
Previous CVD, %	0.6	0.8	1.0	1.0	0.5	10.6	< 0.0001	1.3	0.7	2.4	0.2

Unless stated otherwise the numbers in columns are means ± s.d. P-values represent comparisons with the profile *LowMed.*

### Medication profiles and rapid decline of kidney function

The eGFR slopes ranged from − 18.6 to 17.4, with a median value of − 0.35 (− 1.23 to 0.53) ml/min/1.73 m^2^ per year. About 7.5% of the cohort had a rapid decline in kidney function. Only profiles *ARB* (11.3%, *p* = 0.02) and *HighMed* (12.2%, *p* = 0.004) had higher proportions of women with rapid kidney function decline compared with profile *LowMed* (4.4%). Similarly, in a logistic regression analysis (Table 2), after full adjustment for clinical variables, the odds ratios (ORs) for having rapid decline of kidney function were increased in profile *ARB* (2.75 [1.17, 6.49], *p* = 0.02) and, also in profile *HighMed* (OR 2.55 [1.11, 5.88], *p* = 0.03).

**Table 2:** Multivariable logistic regression analysis for the associations of medication profiles and rapid decline of kidney function.

Profile	Model 1^1^	Model 2^2^	Model 3^3^
	OR (95% CI), *p*	OR (95% CI), *p*	OR (95% CI), *p*
LowMed	1.00	1.00	1.00
Antibacterial	2.04 (1.01, 4.12), 0.05	2.02 (1.00, 4.09), 0.05	1.52 (0.70, 3.31), 0.3
ARB	2.88 (1.26, 6.57), 0.01	2.85 (1.25, 6.52), 0.01	2.75 (1.17, 6.49), 0.02
HighMed	3.12 (1.46, 6.68), 0.003	2.99 (1.36, 6.56), 0.006	2.55 (1.11, 5.88), 0.03
Anti-inflammatory	1.57 (0.77, 3.18), 0.2	1.55 (0.76, 3.15), 0.2	1.49 (0.70, 3.14), 0.3
ACE & Lipids	2.10 (0.82, 5.38), 0.1	2.04 (0.79, 5.72), 0.1	1.73 (0.64, 4.68), 0.3

^1^Unadjusted (n = 1164).

^2^Adjusted for age (n = 1164).

^3^Adjusted for age, systolic BP, waist-to-height ratio, triglycerides, LDL-cholesterol, HbA_1c_, smoking, and eGFR (n = 1060).

Women in profile *ARB* and profile *ACE & Lipids* differed with respect to their kidney outcomes, despite similar clinical characteristics at baseline (Supplementary Table S4), except for age, since women in profile *ARB* were younger (mean age 35.4 vs 40.9 years, *p* = 0.003). Furthermore, there were no differences between the groups concerning the proportions of women (*p* = 0.08) above the BP target range (< 130/80 mmHg) or the purchases of antihypertensive treatment by those out of the target range (*p* = 0.2) (Supplementary Table S5). However, we found differences between these two profiles regarding the proportions of lipid-modifying drug purchases (*p* = 0.03), although there were no differences in the proportions of individuals above the target range of triglycerides (< 1.7 mmol/l) and LDL-cholesterol (< 2.6 mmo/l). In the profile *ARB*, about 63% of the women did not reach their LDL-cholesterol target and only 10% of them had purchased lipid-modifying drugs at baseline, whereas in profile *ACE & Lipids* the numbers were 67% and 27%, respectively. Moreover, although 13% of the women in profile *ARB* and only 7.5% in profile *ACE & Lipids* did not reach their triglycerides target, none of the women in profile *ARB* had purchased lipid-modifying drugs, whereas half of the women in profile *ACE & Lipids* had such medication in their treatment regimen at baseline. Given that prescriptions of RAAS inhibitors and statins are contraindicated during pregnancy, this confounder variable was analyzed separately, and there was no difference in the proportion of women with pregnancy-related clinic visits between profile *ARB* and *ACE & Lipids* (32% vs 22%, *p* = 0.2).

**Figure 2:**
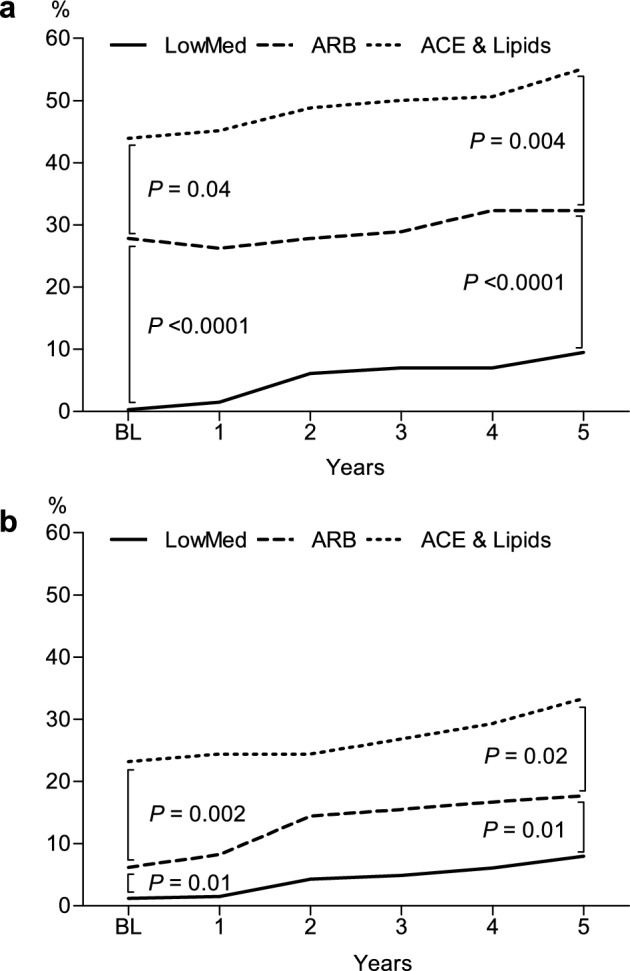
Proportions of women on the renin–angiotensin–aldosterone system (RAAS) inhibitors (**A**) and lipid-lowering drugs (**B**) in profiles *LowMed*, *ARB* and *ACE & Lipids* during the 5-year follow-up.

Of note, the observed baseline differences between the profiles *ARB *and *ACE & Lipids* persisted after 5 years of follow-up (Fig. 2). At baseline, the proportion of women on RAAS inhibitors in profile *ARB* was lower than in profile *ACE & Lipids* (28% vs 44%, *p* = 0.04), moreover, the gap increased 5 years afterwards (32% vs 55%, *p* = 0.004). Regarding lipid-lowering medication purchases, the proportion of women in profile *ARB* was also lower than in profile *ACE & Lipids* at baseline (6% vs 23%, *p* = 0.002), and the gap remained after 5 years (18% vs 33%, *p* = 0.02).

## Discussion

In this study, we characterized six medication profiles from large prescription records in a cohort of women with type 1 diabetes and normal AER, by utilizing data-driven approach to capture the complexity of medication data. Most importantly, we found that two medication profiles, *ARB* and *HighMed*, were associated with a future rapid decline of kidney function. Furthermore, profiles *ARB* and *ACE & Lipids* had different purchases of RAAS inhibitors and lipid-modifying drugs despite similar baseline clinical characteristics, and experienced different kidney outcomes.

Considering the women in profile *HighMed*, a worse kidney outcome was expected as their baseline clinical characteristics were worse compared with profile *LowMed*. However, the women in profile *ARB* experienced a rapid decline of kidney function, that could not be solely explained by baseline clinical characteristics. Comparing these women to the women in profile *ACE & Lipids*, who did not experience a rapid decline of kidney function, the only clinical difference was that women in profile *ARB* were younger. Notwithstanding baseline similarities in BP and lipids between these two profiles, women in profile *ARB* had a lower purchase of RAAS inhibitor and lipid-lowering drugs at baseline that persisted during 5 years of follow-up. Our study raises the question, whether there was an underestimation of the risk of kidney disease in the young women with type 1 diabetes in profile *ARB* due to their young age, and therefore received fewer prescriptions of kidney protective treatments than the older women with similar clinical characteristics in profile *ACE & Lipids.* Hence, the women in profile *ARB* seemed to be less protected from rapid decline of their kidney function. This is especially noteworthy as previous studies have shown that women with type 1 diabetes are less likely to use cardio-protective medications to reduce the risk for coronary heart disease^12^. Evidence from the general population have also shown that young women wait longer than men (at the same age) to be evaluated by a physician and are less likely to have a medication prescription, when admitted to the emergency department due to chest pain^31^.

The impact of both hypercholesterolemia and hypertriglyceridemia on diabetic kidney disease progression is not new knowledge^32^, and the DCCT/EDIC study has also shown that dyslipidemia is an important risk factor for diabetic kidney disease^33^. Of note, about 63% of women in profile *ARB* did not reach their LDL-cholesterol target, but only 10% of them had purchased lipid-modifying drugs at baseline, whereas in profile *ACE & Lipids* the numbers were 67% and 27%, respectively. Importantly, we did not find any evidence that the differences in cardio- and kidney-protective medication purchases between profile *ARB* and *ACE & Lipids* are driven by pregnancy-related discontinuation of RAAS inhibitors or lipid-lowering medications. However, we cannot completely exclude the possibility that a higher percentage of women in profile *ARB* discontinued their medication because they were planning to become pregnant. Thus, our study suggests that there might be a subgroup of young women with type 1 diabetes, who might need special attention to achieve guideline targets to prevent future loss of kidney function. However, further studies are needed to estimate a possible care gap in cardio- and kidney-protective medication use by age and sex among type 1 diabetes individuals.

The strength of our study includes its carefully clinical characterization of a large cohort of women with type 1 diabetes, including information on all their outpatient prescriptions. Moreover, using the data-driven SOM approach allowed us to identify medication profiles directly from the drug register data without any assumptions on the number of existing profiles. There are limitations in our study that need to be acknowledged. First, medication profiles were constructed from cross-sectional data. Therefore, it might be possible that guidelines regarding the use of medications have changed over time. However, the median FinnDiane baseline years did not differ significantly in women with the profiles of interest. Although all our models (unadjusted, minimally adjusted and fully adjusted) support our conclusions and the FinnDiane participants have been carefully examined, residual confounding cannot be completely excluded. In addition, although eGFR slopes were calculated during the long follow-up, our model did not consider non-linear patterns of eGFR decline. There is, however, evidence that eGFR slopes are predominantly linear in individuals with type 1 diabetes^34^. One such non-linear pattern could arise at the initiation of treatment with ARBs or ACE inhibitors as they might lead to an acute fall in eGFR, but also a long-term slower decline in renal function^34^. Nevertheless, our inclusion criteria were carefully chosen to minimize such effects on the calculation of the eGFR slopes by requiring at least two years of follow-up and three eGFR measures. However, we cannot categorically exclude the possibility of misclassification with regards to rapid kidney function decline. Here we present findings from cross-sectional, observational data, and cannot address questions regarding causality with this study design. Finally, it is important that our study results should be replicated in other cohorts.

In conclusion, medication profiles created solely from a large prescription register database revealed differences in women with type 1 diabetes and normal AER. Two baseline medication profiles (*ARB* and *HighMed*) were associated with future rapid decline of kidney function. Highly medicated women in profile *HighMed* had already worse clinical characteristics at baseline, which might explain their association with rapid kidney function decline. Importantly, we found a group of younger women (profile *ARB*) that showed some potential evidence that their risk of future decline of kidney function could be addressed by earlier pharmacological interventions, better surveillance of achieving guideline targets and earlier intervention if targets are not achieved. Our results highlight the importance of close follow-up of younger women with type 1 diabetes that are usually considered to be at low-risk for a rapid decline of kidney function.

### Supplementary Information

Supplementary Information.

## Data Availability

Individual-level data for the study participants are not publicly available because of the restrictions due to the study consent provided by the participant at the time of data collection. The access to individual-level data, which is subject to local regulations, can be obtained upon reasonable request by contacting the FinnDiane Study Group board (manage@finndiane.fi). Upon approval, analysis needs to be performed on a user-specific local server (with protected access) and requires the applicant to sign non-disclosure and privacy agreements and comply with the General Data Protection Regulation.
