# Supplementary material for: Medication profiling in women with type 1 diabetes highlights the importance of adequate, guideline-based treatment in low-risk groups
Source: Sci Rep. 2023 Oct 19;13:17893. doi: 10.1038/s41598-023-44695-2 (PMC10587128; doi:10.1038/s41598-023-44695-2)

Supplementary Material

Raija Lithovius, Stefan Mutter, Erika B. Parente, Ville-Petteri Mäkinen, Erkka Valo, Valma Harjutsalo and Per-Henrik Groop, MD, DMSc; on behalf of the FinnDiane Study Group. **Medication profiling in women with type 1 diabetes highlights the importance of adequate, guideline-based treatment in low-risk groups**

Contents

Supplementary Table S1. Physicians and nurses at each of the FinnDiane centers participating in patient recruitment and characterization 2

Supplementary Table S2. The 3. level of ATC codes for the 37 pharmacological subgroups 4

Supplementary Table S3. Counts of the women who had purchased drugs from each pharmacological subgroup at baseline 5

Supplementary Table S4. Baseline characteristics of women in the medication profiles *ARB* and *ACE & Lipids* 6

Supplementary Table S5. Blood pressure, LDL cholesterol or triglycerides above the targets, based on the ADA's diabetes guidelines and pharmacological treatment 7

Supplementary Fig. S1. Sematic illustration of the study procedure........ 8

Supplementary Table S1. Physicians and nurses at each of the FinnDiane centers participating in patient recruitment and characterization

The Finnish Diabetic Nephropathy Study Center	Physicians and nurses
Anjalankoski Health Center	S.Koivula, T.Uggeldahl
Central Finland Central Hospital, Jyväskylä	T.Forslund, A.Halonen, A.Koistinen, P.Koskiaho, M.Laukkanen, J.Saltevo, M.Tiihonen
Central Hospital of Åland Islands, Mariehamn	M.Forsen, H.Granlund, A.-C.Jonsson, B.Nyroos
Central Hospital of Kanta-Häme, Hämeenlinna	P.Kinnunen, A.Orvola, T.Salonen, A.Vähänen
Central Hospital of Kymenlaakso, Kotka	R.Paldanius, M.Riihelä, L.Ryysy
Central Hospital of Länsi-Pohja, Kemi	H.Laukkanen, P.Nyländen, A.Sademies
Central Ostrobothnian Hospital District, Kokkola	S.Anderson, B.Asplund, U.Byskata, P.Liedes, M.Kuusela, T.Virkkala
City of Espoo Health Center:	
Espoonlahti	A.Nikkola, E.Ritola
Tapiola	M.Niska, H.Saarinen
Samaria	E.Oukko-Ruponen, T.Virtanen
Viherlaakso	A.Lyytinen
City of Helsinki Health Center:	
Puistola	H.Kari, T.Simonen
Suutarila	A.Kaprio, J.Kärkkäinen, B.Rantaeskola
Töölö	P.Kääriäinen, J.Haaga, A-L.Pietiläinen
City of Hyvinkää Health Center	S.Klemetti, T.Nyandoto, E.Rontu, S.Satuli-Autere
City of Vantaa Health Center:	
Korso	R.Toivonen, H.Virtanen
Länsimäki	R.Ahonen, M.Ivaska-Suomela, A.Jauhiainen
Martinlaakso	M.Laine, T.Pellonpää, R.Puranen
Myyrmäki	A.Airas, J.Laakso, K.Rautavaara
Rekola	M.Erola, E.Jatkola
Tikkurila	R.Lönnblad, A.Malm, J.Mäkelä, E.Rautamo
Heinola Health Center	P.Hentunen, J.Lagerstam
Helsinki University Central Hospital, Department of Medicine, Division of Nephrology	M.Feodoroff, D.Gordin, O.Heikkilä, K.Hietala, J.Fagerudd, M.Korolainen, L.Kyllönen, J.Kytö, S.Lindh, K.Pettersson-Fernholm, M.Rosengård-Bärlund, A.Sandelin, L.Thorn, J.Tuomikangas, T.Vesisenaho, J.Wadén
Herttoniemi Hospital, Helsinki	V.Sipilä
Hospital of Lounais-Häme, Forssa	T.Kalliomäki, J.Koskelainen, R.Nikkanen, N.Savolainen, H.Sulonen, E.Valtonen
Hyvinkää Hospital	L. Norvio, A.Hämäläinen
Iisalmi Hospital	E.Toivanen
Jokilaakso Hospital, Jämsä	A.Parta, I.Pirttiniemi
Jorvi Hospital, Helsinki University Central Hospital	S.Aranko, S.Ervasti, R.Kauppinen-Mäkelin, A.Kuusisto, T.Leppälä, K.Nikkilä, L.Pekkonen
Jyväskylä Health Center, Kyllö	K.Nuorva, M.Tiihonen
Kainuu Central Hospital, Kajaani	S.Jokelainen, K.Kananen, M.Karjalainen, P.Kemppainen, A-M.Mankinen, A.Reponen M.Sankari
Kerava Health Center	H.Stuckey, P.Suominen
Kirkkonummi Health Center	A.Lappalainen, M.Liimatainen, J.Santaholma
Kivelä Hospital, Helsinki	A.Aimolahti, E.Huovinen
Koskela Hospital, Helsinki	V.Ilkka, M.Lehtimäki
Kotka Health Center	E.Pälikkö-Kontinen, A.Vanhanen
Kouvola Health Center	E.Koskinen, T.Siitonen
Kuopio University Hospital	E.Huttunen, R.Ikäheimo, P.Karhapää, P.Kekäläinen, M.Laakso, T.Lakka, E.Lampainen, L.Moilanen, S. Tanskanen, L.Niskanen, U.Tuovinen, I.Vauhkonen, E.Voutilainen
Kuusamo Health Center	T.Kääriäinen, E.Isopoussu
Kuusankoski Hospital	E.Kilkki, I.Koskinen, L.Riihelä
Laakso Hospital, Helsinki	T.Meriläinen, P.Poukka, R.Savolainen, N.Uhlenius
Lahti City Hospital	A.Mäkelä, M.Tanner
Lapland Central Hospital, Rovaniemi	L.Hyvärinen, K.Lampela, S.Pöykkö, T.Rompasaari, S.Severinkangas, T.Tulokas
Lappeenranta Health Center	P. Erola, L.Härkönen, P.Linkola, T.Pekkanen, I.Pulli, E.Repo
Lohja Hospital	T.Granlund, K.Hietanen, M.Porrassalmi, M.Saari, T.Salonen, M.Tiikkainen,
Länsi-Uusimaa Hospital, Tammisaari	I.-M.Jousmaa, J.Rinne
Loimaa Health Center	A.Mäkelä, P.Eloranta
Malmi Hospital, Helsinki	H.Lanki, S.Moilanen, M.Tilly-Kiesi
Mikkeli Central Hospital	A.Gynther, R.Manninen, P.Nironen, M.Salminen, T.Vänttinen
Mänttä Regional Hospital	I.Pirttiniemi, A-M.Hänninen
North Karelian Hospital, Joensuu	U-M.Henttula, P.Kekäläinen, M.Pietarinen, A.Rissanen, M.Voutilainen
Nurmijärvi Health Center	A.Burgos, K.Urtamo
Oulaskangas Hospital, Oulainen	E.Jokelainen, P-L.Jylkkä, E.Kaarlela, J.Vuolaspuro

Oulu Health Center	L.Hiltunen, R.Häkkinen, S.Keinänen-Kiukaanniemi
Oulu University Hospital	R.Ikäheimo
Päijät-Häme Central Hospital	H.Haapamäki, A.Helanterä, S.Hämäläinen, V.Ilvesmäki, H.Miettinen
Palokka Health Center	P.Sopanen, L.Welling
Pieksämäki Hospital	V.Sevtsenko, M.Tamminen
Pietarsaari Hospital	M-L.Holmbäck, B.Isomaa, L.Sarelin
Pori City Hospital	P.Ahonen, P.Merisalo, E.Muurinen, K.Sävelä
Porvoo Hospital	M.Kallio, B.Rask, S.Rämö
Raahe Hospital	A.Holma, M.Honkala, A.Tuomivaara, R.Vainionpää
Rauma Hospital	K.Laine, K.Saarinen, T.Salminen
Riihimäki Hospital	P.Aalto, E.Immonen, L.Juurinen
Salo Hospital	A.Alanko, J.Lapinleimu, P.Rautio, M.Virtanen
Satakunta Central Hospital, Pori	M.Asola, M.Juhola, P.Kunelius, M.-L.Lahdenmäki, P.Pääkkönen, M.Rautavirta
Savonlinna Central Hospital	T.Pulli, P.Sallinen, M.Taskinen, E.Tolvanen, T.Tuominen, H.Valtonen, A.Vartia, S-L. Viitanen
Seinäjoki Central Hospital	O.Antila, E.Korpi-Hyövälti, T.Latvala, E.Leijala, T.Leikkari, M.Punkari, N.Rantamäki, H.Vähävuori
South Karelia Central Hospital, Lappeenranta	T.Ensala, E.Hussi, R.Härkönen, U.Nyholm, J.Toivanen
Tampere Health Center	A.Vaden, P.Alarotu, E.Kujansuu, H.Kirkkopelto-Jokinen, M.Helin, S.Gummerus, L.Calonius, T.Niskanen, T.Kaitala, T.Vatanen
Tampere University Hospital	P. Hannula, I.Ala-Houhala, R.Kannisto, T.Kuningas, P.Lampinen, M.Määttä, H.Oksala, T.Oksanen, A.Putila, H.Saha, K.Salonen, H.Tauriainen, S.Tulokas
Tiirismaa Health Center, Hollola	T.Kivelä, L.Petlin, L.Savolainen
Turku Health Center	A.Artukka, I.Hämäläinen, L.Lehtinen, E.Pyysalo, H.Virtamo, M.Viinikkala, M.Vähätalo
Turku University Central Hospital	K.Breitholz, R.Eskola, K.Metsärinne, U.Pietilä, P.Saarinen, R.Tuominen, S.Äyräpää
Vaajakoski Health Center	K.Mäkinen, P.Sopanen
Valkeakoski Regional Hospital	S.Ojanen, E.Valtonen, H.Ylönen, M.Rautiainen,T.Immonen
Vammala Regional Hospital	I.Isomäki, R.Kroneld, L.Mustaniemi, M.Tapiolinna-Mäkelä
Vaasa Central Hospital	S.Bergkulla, U.Hautamäki, V-A.Myllyniemi, I.Rusk

Supplementary Table S2. The 3rd level of the Anatomical Therapeutic Chemical (ATC) classification system codes for the 37 pharmacological subgroups with at least 2% purchases at baseline. Insulins (ATC C10A) were not included.

	ATC	Purchases at baseline	Percentages
1	M01A	335	23.54
2	J01C	257	18.06
3	J01D	237	16.65
4	J01F	174	12.23
5	J02A	174	12.23
6	H03A	151	10.61
7	R06A	147	10.33
8	J01A	141	9.91
9	N06A	124	8.71
10	C09A	110	7.73
11	R01A	106	7.45
12	C10A	103	7.24
13	R05F	95	6.68
14	G03C	93	6.54
15	H04A	85	5.97
16	A02B	84	5.90
17	C07A	73	5.13
18	G03F	68	4.78
19	M03B	68	4.78
20	G03D	67	4.71
21	R03A	67	4.71
22	R01B	64	4.50
23	D07A	63	4.43
24	J01M	61	4.29
25	J01E	60	4.22
26	R03B	59	4.15
27	N05C	48	3.37
28	D01A	43	3.02
29	G01A	41	2.88
30	G03H	40	2.81
31	N05B	39	2.74
32	R05D	38	2.67
33	C01D	35	2.46
34	N03A	33	2.32
35	C09C	32	2.25
36	G03A	31	2.18
37	S01G	30	2.11

Supplementary Table S3. Counts of the women who had purchased drugs from each pharmacological subgroup at baseline. *P*-values represent comparisons with the study average.

**Profile**	***LowMed***		***Anti-bacterial***		***ARB***		***HighMed***		***Anti-inflammatory***		***ACE & Lipids***		**All**
N	329		241		97		123		292		82		1164
**Drug group (ATC-code)**	n (%)	*p*	n (%)	*p*	n (%)	*p*	n (%)	*p*	n (%)	*p*	n (%)	*p*	n (%)
ACE inhibitors, plain (C09A)	0 (0)	<0.0001	10 (4.1)	0.07	5 (5.1)	0.5	23 (18.7)	<0.0001	17 (5.8)	1.0	35 (42.7)	<0.0001	90 (7.7)
Angiotensin receptor blockers, plain (C09C)	0 (0)	0.01	0 (0)	0.01	23 (23.7)	<0.0001	2 (1.6)	1.0	0 (0)	0.02	0 (0)	0.4	25 (2.1)
Lipid-modifying agents, plain (C10A)	4 (1.2)	0.0002	6 (2.5)	0.02	6 (6.2)	1.0	25 (20.3)	<0.0001	17 (5.8)	0.7	19 (23.2)	<0.0001	77 (6.6)
Anti-inflammatory and antirheumatic products (M01A)	0 (0)	<0.0001	38 (15.8)	0.007	23 (23.7)	1.0	46 (37.4)	0.002	172 (58.9)	<0.0001	0 (0)	<0.0001	279 (24.0)
Other beta-lactam anti-bacterials, cephalosporins (J01D)	0 (0)	<0.0001	101 (41.9)	<0.0001	22 (22.7)	0.1	19 (15.4)	1.0	42 (14.4)	0.6	2 (2.4)	0.002	186 (16.0)
Antidepressants (N06A)	11 (3.3)	0.002	9 (3.7)	0.01	8 (8.2)	1.0	43 (35.0)	<0.0001	17 (5.8)	0.2	12 (14.6)	0.1	100 (8.6)

Supplementary Table S4. Baseline characteristics of women in the medication profiles *ARB* and *ACE & Lipids*.
Data are presented as mean (± s.d.), median (with q1, q3) or %

**Profile**	***ARB***	***ACE & Lipids***	***p***
N	97	82	
Age (years)	35.4 ± 13.4	40.9 ± 10.9	0.003
Duration of diabetes (years)	18.4 ± 13.3	21.7 ± 11.7	0.08
Median age at onset of diabetes (years)	14.2 (10.0 – 23.7)	17.9 (10.9 – 25.7)	0.09
HbA1c (%)	8.4 ± 1.3	8.1 ± 1.4	0.3
HbA1c (mmol/mol)	68 ± 14	65 ± 15	0.3
Systolic BP (mmHg)	128 ± 17	133 ± 18	0.08
Diastolic BP (mmHg)	77 ± 9	80 ± 10	0.06
BMI (kg/m^2)	24.6 ± 3.2	25.0 ± 3.9	0.5
Waist-to-height ratio	0.47 ± 0.05	0.49 ± 0.07	0.2
Total cholesterol (mmol/l)	4.97 ± 0.91	5.08 ± 0.95	0.4
LDL cholesterol (mmol/l)	2.96 ± 0.84	3.11 ± 0.94	0.3
HDL cholesterol (mmol/l)	1.54 ± 0.39	1.54 ± 0.47	1.0
Median triglycerides (mmol/l)	0.88 (0.68 – 1.26)	0.88 (0.65 – 1.15)	0.3
Median CRP (mg/l)	2.14 (1.34 – 5.12)	2.17 (1.28 – 4.17)	0.2
History of smoking (%)	33.7	33.3	1.0
Median baseline FinnDiane visit (years)	2001 (2000 –2002)	2000 (1999 – 2002)	0.2
Median eGFR (ml/min/1.73 m^2)	103 (87 – 116)	102 (84 – 111)	0.4
Previous CVD (%)	1.0	2.4	0.6
Pregnancy-related clinical visits 1 year before or after the baseline (%)	32.0	22.0	0.2

Supplementary Table S5. Blood pressure, LDL cholesterol or triglycerides above the targets, based on the American Diabetes Association's (ADA's) diabetes guidelines and pharmacological treatment (i.e. lipid-modifying or antihypertensive drugs)

**At baseline**	**Blood pressure ≥130/80 mmHg (%)**				
	**Profile *ARB***		**Profile *ACE & Lipids***		***p***
	62.6		76.3		0.08
	No	Yes	No	Yes	
Antihypertensive drugs[1] n (%)	32 (56.1)	25 (43.9)	26 (42.6)	35 (57.4)	0.2
RAAS[2] n (%)	34 (59.6)	23 (40.4)	29 (47.5)	32 (52.5)	0.3
**At baseline**	**LDL cholesterol ≥2.6 mmol/l (%)**				
	**Profile *ARB***		**Profile *ACE & Lipids***		***P***
	62.8		67.1		0.7
	No	Yes	No	Yes	
Lipid-modifying drugs[3] n (%)	53 (89.8)	6 (10.2)	40 (72.7)	15 (27.3)	0.03
**At baseline**	**Triglycerides ≥1.7 mmol/l (%)**				
	**Profile *ARB***		**Profile *ACE & Lipids***		***P***
	12.8		7.4		0.3
	No	Yes	No	Yes	
Lipid-modifying drugs[3] n (%)	12 (100)	0 (0)	3 (50.0)	3 (50.0)	0.02

[1]Antihypertensive drugs (ATC-codes C02A, C02C, C02D, C03A, C03B, C03C, C03D, C03E, C07A, C07B, C07F, C08C, C08D, C09A, C09B, C09C, C09D, C09X)
[2]Agents acting on the renin-angiotensin system (ATC-codes C09A, C09B, C09C, C09D, C09X)
[3]Lipid-modifying drugs (C10A, C10B)

Supplementary Fig. S1. Sematic illustration of the study procedure. Medication profiles were created in women with normal albumin execration rate (AER) (N=1,164) by utilising data-driven method, so called self-organising maps (SOM). The outcome of the study was rapid decline of kideny function, defined as an estimated glomerular filtration rate (eGFR) slope steeper than -3 ml/min/1.73 m^2 per year.

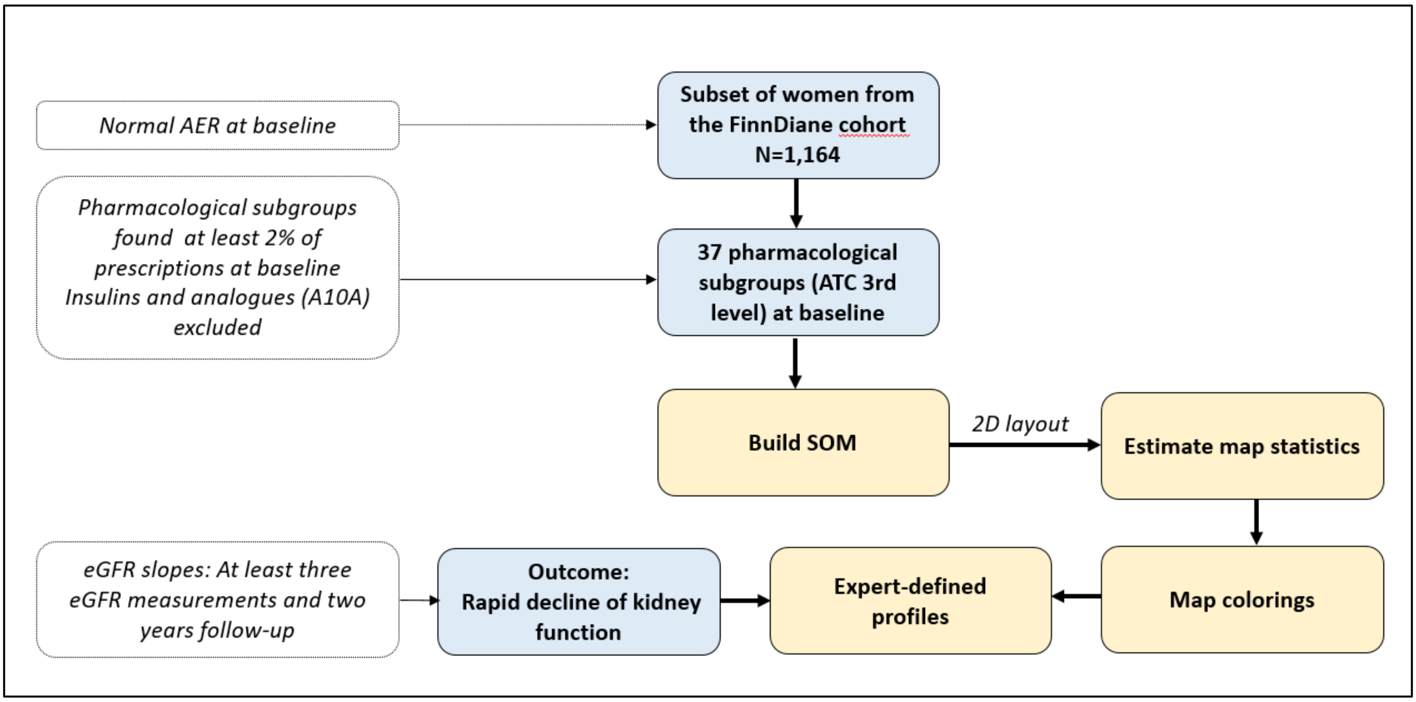

Supplement: Supplementary file 1 — Supplementary Information. [file 41598_2023_44695_MOESM1_ESM.pdf]
